# **Irish national real-world analysis of the clinical and economic impact of 21-gene oncotype DX® testing in early-stage, 1**-**3 lymph node-positive, oestrogen receptor-positive, HER2-negative, breast cancer**

**DOI:** 10.1007/s10549-024-07486-5

**Published:** 2024-10-04

**Authors:** I. M. Browne, R. A. McLaughlin, C. S. Weadick, S. O’Sullivan, L. M. McSorley, D. K. Hadi, S. J. Millen, M. J. Higgins, J. P. Crown, R. S. Prichard, D. P. McCartan, A. DK. Hill, R. M. Connolly, S. A. Noonan, D. O’Mahony, C. Murray, C. O’Hanlon-Brown, B. T. Hennessy, C. M. Quinn, C. M. Kelly, S. O’Reilly, P. G. Morris, J. M. Walshe

**Affiliations:** 1 Department of Medical Oncology, St Vincent’s University Hospital, Dublin 4, Ireland; 2 Department of Medical Oncology, Beaumont Hospital, Beaumont, Dublin, Ireland; 3 Department of Medical Oncology, Cork University Hospital, Cork, Ireland; 4 Department of Medical Oncology, Mater Misericordiae University Hospital, Dublin 7, Ireland; 5 Department of Medical Oncology, St James’s Hospital, Dublin 8, Ireland; 6 Exact Sciences UK Ltd, London, UK; 7 Department of Surgery, St. Vincent’s University Hospital, Dublin, Ireland; 8 Department of Surgery, Royal College of Surgeons, Dublin, Ireland; 9 Cancer Research @ UCC, University College Cork, Cork, Ireland; 10 Cork University Hospital/University College Cork Cancer Centre, Cork, Ireland; 11 Department of Medical Oncology, Bon Secours Hospital, Cork, Ireland; 12 Department of Pathology, St. Vincent’s University Hospital, Dublin 4, Ireland; 13 University College Dublin, Dublin, Ireland; 14 Cancer Trials Ireland, Dublin, Ireland

**Keywords:** Breast cancer, Node-positive, Oncotype DX recurrence score, Decision impact, Economic impact

## Abstract

**Purpose:**

The treatment landscape of Oestrogen receptor-positive (ER-positive) breast cancer is evolving, with declining chemotherapy use as a result of Oncotype DX Breast Recurrence Score® testing. Results from the SWOG S1007 RxPONDER trial suggest that adjuvant chemotherapy may benefit some premenopausal women with ER-positive, HER2-negative disease with 1–3 positive lymph nodes (N1), and a Recurrence Score® (RS) of ≤ 25. Postmenopausal women with similar characteristics did not benefit from adjuvant chemotherapy. We examine the clinical and economic impact of Oncotype DX® testing on treatment decisions in patients with N1 disease in Ireland using real world data.

**Methods:**

From March 2011 to October 2022, a retrospective, cross-sectional observational study was performed of patients with ER-positive, HER2-negative N1 breast cancer, who had Oncotype DX testing across 5 of Ireland’s largest cancer centres. Patients were classified into low risk (RS 0–13), intermediate risk (RS 14–25) and high risk (RS > 25). Data were collected via electronic patient records. Information regarding costing was provided primarily by pre-published sources.

**Results:**

A total of 828 N1 patients were included in this study. Post Oncotype DX testing, 480 patients (58%) were spared chemotherapy. Of the patients who had a change in chemotherapy recommendation based on Oncotype DX testing, 271 (56%), 205 (43%), 4 (1%) had a RS result of 0–13, 14–25 and > 25 respectively. Use of Oncotype DX testing was associated with a 58% reduction in chemotherapy administration overall. This resulted in estimated savings of over €6 million in treatment costs. Deducting the assay cost, estimated net savings of over €3.3 million were achieved. Changes in the ordering demographics of Oncotype DX tests were identified after RxPONDER data were presented, with increased testing in women ≥ 50 years and a reduction in proportion of tests ordered for women < 50 years.

**Conclusion:**

Between 2011 and 2022, assay use resulted in a 58% reduction in chemotherapy administration and net savings of over €3.3 million.

**Supplementary Information:**

The online version contains supplementary material available at 10.1007/s10549-024-07486-5.

## Background

Breast cancer is the most-commonly diagnosed female cancer and second most common cancer overall with almost 4000 cases diagnosed in Ireland annually [1]. With the introduction of mammographic screening, there has been a stage shift over time with an increase in the relative number of early-stage breast cancers, accounting for approximately 80% of breast cancers in Ireland [2]. Approximately one third of women with newly diagnosed ER-positive, HER2-negative breast cancer have positive lymph nodes at presentation [3]. The goal for this patient cohort is to provide an individualised treatment plan. While pathological and clinical features including patient age, ER-status, tumour size, lymph node involvement, histological subtype, grade and Ki67 have traditionally been used to guide treatment decisions, these are insufficient parameters to predict who will experience benefit from chemotherapy and who can safely be spared the side effects, leading to overtreatment [4]. Lymph node involvement, particularly, is an important prognostic factor in early-stage breast cancer and is associated with an increased risk of local and distant disease recurrence, which in turn directly affects mortality [5–7]. However, lymph node involvement does not seem to influence the absolute benefit of chemotherapy in terms of 5-year invasive disease-free survival (iDFS) [8]. As such, determining the optimal course of treatment for this subgroup is of huge importance. Multigene expression profiling when used in addition to clinical-pathological factors has revolutionised the adjuvant treatment of ER-positive, HER2-negative breast cancer patients [9–15].

The Oncotype DX® test (Genomic Health, Inc., Redwood City CA, USA) is a multigene assay which has been prospectively validated in women with ER-positive, HER2-negative breast cancer in the TAILORx (node negative) and RxPONDER (1–3 lymph nodes positive) trials [13, 14]. It is a reverse transcriptase polymerase chain reaction-based assay which quantifies the expression of 21 genes (16 cancer-related, 5 reference genes) in tumour tissue. These results are combined and generate a Recurrence Score® (RS) value from 0 to 100 which reflects underlying tumour biology, with higher scores indicating a worse prognosis and predicting the likelihood of chemotherapy benefit [16].

Until recently international guidelines had recommended the consideration of chemotherapy for the majority of patients with ER-positive, HER2-negative tumours with 1–3 positive lymph nodes [17, 18]. Similar to the node-negative setting, retrospective data suggested that only some patients benefit from adjuvant chemotherapy [19, 20]. The potential benefits of Oncotype DX testing in N1 patients were first suggested in a prospective-retrospective analysis of the phase 3 SWOG 8814 trial which identified no statistically significant benefit with the addition of chemotherapy to anti-hormonal therapy in terms of iDFS and overall survival (OS) for patients with a RS < 18 [19]. Similar results in a retrospective analysis of the Clalit Health registry supported the use of endocrine therapy alone in this population [20]. These studies provided the basis for the pivotal phase 3 RxPONDER trial which evaluated the prognostic and predictive role of Oncotype DX testing among women with ER-positive, HER2-negative, N1 disease [14]. In this trial, patients were classified into low risk (RS 0–13), intermediate risk (RS 14–25), and high risk (RS > 25), and patients with a RS from 0 to 25 were included. Among postmenopausal patients with a RS ≤ 25, recurrence rates were comparable regardless of the addition of chemotherapy to anti-hormonal therapy. In contrast, in premenopausal women, a benefit was seen for chemo-endocrine therapy vs. endocrine therapy, with an iDFS at 5 years of 89.0% with endocrine-only therapy and 93.9% with chemo-endocrine therapy (HR, 0.60; 95% CI, 0.43 to 0.83; *p* = 0.002), and a similar increase in distant relapse-free survival. Notably the relative chemotherapy benefit did not increase as the recurrence score increased [14].

To date, a limited number of real-world decision impact studies suggest that Oncotype DX testing in N1 patients leads to a significant reduction in adjuvant chemotherapy administration, alongside a favourable budget impact [21–25].

Ireland was one of the first public healthcare systems globally to approve reimbursement for Oncotype DX testing in node-negative and N1 patients. The objectives of this study were to investigate the real-world experience of both the clinical and economic impact of Oncotype DX testing in Irish ER-positive, HER2-negative breast cancer patients with 1–3 positive lymph nodes. Furthermore, as our patients had access to Oncotype DX testing pre- and post-presentation of the RxPONDER data, we also wanted to review the impact those results had on Oncotype DX ordering demographics over those two periods.

## Methods

### Study design, data source and study population

A multi-site, retrospective, cross-sectional observational study was conducted across five of Ireland’s Cancer Centres. All patients with ER-positive, HER2-negative, N1 breast cancer who underwent Oncotype DX testing between March 2011 and October 2022 were identified. Once identified, electronic records were used to collect clinical data. These data were supplemented by a review of paper medical notes as required. All patients underwent surgery to remove the primary breast tumour with either sentinel lymph node biopsy or axillary lymph node dissection. Patients with micro-metastases were included; however, isolated tumour cells were excluded.

Clinical data collected for each patient included age at diagnosis, multifocality of disease, tumour size, tumour grade, tumour morphology, presence or absence of lymphovascular invasion (LVI), number of positive nodes, stage (AJCC 8th edition), and RS result. Treatment details included surgery (breast conserving surgery or mastectomy), and if adjuvant radiotherapy was administered. We also included systemic treatment recommendations’ post-Oncotype DX testing, recorded what chemotherapy and anti-hormonal therapy, the patient went on to receive, and for premenopausal women whether ovarian function suppression was given.

In Ireland, the Oncotype DX test is not undertaken in patients who are not considered candidates for adjuvant chemotherapy for clinical reasons (for example, due to the patient’s age, health status or menopausal status), and may not be undertaken in some patients for pathological reasons (for example, low tumour grade or small tumour size) [26]. A survey of Irish Breast Oncologists provided the assumption that all patients in our study with N1 disease who underwent Oncotype DX testing would be recommended adjuvant chemotherapy without testing. Using RxPONDER risk groupings, patients were classified into low risk (RS 0–13), intermediate risk (RS 14–25) and high risk (RS > 25).

A standardised excel data collection template was used at each site, with all data de-identified to protect patient confidentiality. The completed excel records from each site were merged onto the primary database for analysis. This database was standardised with uniform nomenclature to aid subsequent statistical analysis. Datalock and submission for statistical analysis were 26/10/22.

### Statistical analysis

IBM SPSS (version 28) was the statistical software used. Descriptive statistics (mean, median and standard deviation for continuous variables; frequency and proportion for categorical variables) were employed and are compared using a t test or a chi-square test, as appropriate.

### Decision impact analysis

In order to facilitate analysis of the impact of Oncotype DX testing on chemotherapy administration and allow the comparison of non-Oncotype DX-guided practice with Oncotype DX-guided practice, a pre-Oncotype DX testing treatment recommendation was assigned to each patient in the dataset. For this analysis in N1 patients, following a survey of Irish Breast Oncologists, a decision was made that in the absence of Oncotype DX testing, standard practice for all patients with N1 disease in our dataset, including those with micro-metastases, would be to recommend adjuvant chemotherapy.

The overall change in the treatment recommendation based on Oncotype DX testing was determined, alongside the net % reduction in adjuvant chemotherapy use.

### Budget impact analysis

A simple budget impact model was employed for this analysis, using the calculated net % reduction in adjuvant chemotherapy usage due to Oncotype DX testing. This impact model compared the cost of Oncotype DX testing with the net savings in chemotherapy-related costs resulting from its use. This analysis was primarily conducted from a third-party payer perspective, which reflects Ireland’s national public health system financing structure funded by public finances accrued by taxation. A secondary analysis of societal budget impact was also conducted. Scenario analyses were performed by varying the assumed net reduction in chemotherapy use due to Oncotype DX testing, chemotherapy-related costs and cost of Oncotype DX testing by ± 25%.

### Treatment cost analysis (third-party payer perspective)

A cost analysis to assess the financial impact of Oncotype DX testing was performed primarily using previously published sources. Chemotherapy day-case costs were sourced from the National Healthcare Pricing Office’s Activity Based Funding (ABF) 2022 Admitted Patient Price List [27], and the weighted average chemotherapy cost per cycle was multiplied by the average number of cycles. The average number of cycles was calculated based on the distribution of chemotherapy regimens for N1 patients sourced from the study dataset.

Average granulocyte-colony stimulating factor (G-CSF) costs were calculated by multiplying the cost per chemotherapy cycle of G-CSF by the average number of chemotherapy cycles by the proportion of patients assumed to receive G-CSF. To account for changes in the cost of G-CSF over time, different G-CSF cost assumptions were used for the period 2011–2020 (€1,246.25 per cycle based on Smyth et al. 2015) and 2021–2022 (€976.78 based on the 2019 high-tech medicines published price list, to reflect the study periods prior to and after reporting of the RxPONDER study results [28] [29]. To estimate the cost per cycle of G-CSF for the overall study time period (€1,158.05), a weighted average of the two G-CSF cost estimates was applied, according to the number of patients receiving chemotherapy in each time period. The 2019 figure was used for a subgroup analysis of patients tested after the RxPONDER study data reported. The manufacturers’ list price for the Oncotype DX test in Ireland (€3180) was assumed for the analysis [30].

Adverse event management costs were estimated based on a weighted average of reported incidence of grade 3/4 adverse events for FEC60 and FEC-D from the TACT trial [31], and unit costs for the treatment of each adverse event. Unit prices for treating the identified adverse events were sourced from various references including ABF 2023 price list for relevant DRG codes [27], Carney 2018—HSE-casemix [32] (average cost for an OPD attendance), and Hinde et al. Health Policy and Technology 2019 [33]. The consumer price index (CPI) for Ireland for the domain ‘Health’ was used to inflate historic prices to current year values. Assumed adverse event cost was €2,282.00 per patient undergoing chemotherapy (Table 1).

**Table 1:** Total cost of chemotherapy used for budget impact analysis (Third-party payer perspective)

Chemotherapy, pharmacy, Administration and monitoring costs	G-CSF treatment	Treatment of adverse events resulting from chemotherapy	Total
€3,906.43 (€617*1 + €572*5.8)	€5,821.72	€2282.00	€12,010.16

### Societal chemotherapy cost analysis

The estimated societal costs of chemotherapy were based on UK cost estimates from a report published by the University of East Anglia, which estimated annual societal costs associated with chemotherapy for early breast cancer in the UK using an incidence-based-cost-of-illness model, and interviews with stakeholders, which included women who had chemotherapy and medical practitioners involved in breast cancer care [34]. Total societal costs of chemotherapy treatment per patient (£103,319) were calculated based on the sum of estimated lost productivity costs due to early mortality (£64,937), long-term work absenteeism (£32,857), short-term work absenteeism (£3,425), caregiver productivity losses (£1,000), and patient/caregiver out-of-pocket costs (£1,100). Secondary malignancy costs were excluded from this sum to avoid double-counting productivity losses from mortality.

For the current analysis, Sterling estimates were converted to Euro costs based on an exchange rate of 1 GBP = 1.17025 EUR on 14/06/2023 to give a total societal cost excluding treatment costs borne by the healthcare system of €120,909.06 per patient and €132,919.22 per patient including treatment costs borne by the healthcare system (Table 2).

**Table 2:** Total cost of chemotherapy from an overall societal perspective

Average total treatment costs borne by healthcare system per patient receiving chemotherapy treatment (overall study period)	Average total productivity losses per patient receiving chemotherapy treatment (including early mortality, short- and long-term work absenteeism and caregiver productivity losses)	Average patient/caregiver out of pocket costs per patient receiving chemotherapy treatment	Average total societal cost of chemotherapy per patient receiving chemotherapy treatment (excluding costs borne by healthcare system)	Average total societal cost of chemotherapy per patient receiving chemotherapy treatment (including costs borne by healthcare system)
€12,010.16	€119,621.78	€1,287.28	€120,909.06	€132,919.22

## Results

### Patient demographics

A total of 828 patients with ER-positive, HER2-negative, N1 breast cancer underwent Oncotype DX testing and were suitable for inclusion in this analysis. Patient and tumour characteristics are summarised in Table 3. Mean patient age was 58 years (range 22–81). One hundred and seventy-one patients (21%) were < 50 years with a mean age of 44, and 657 (79%) patients were aged ≥ 50 with a mean age of 61 years.

**Table 3:** Clinical characteristics and treatment received by 21-gene recurrence score

	Total (*n*)	RS 0–13 (%)	RS 14–25 (%)	RS > 25
Overall	828	319 (38)	396 (48)	113 (14)
Mean age (range)	58 (22–81)	58 (28–81)	57 (31–81)	57 (22–79)
Median age	57	59	57	57
Histology				
IDC	639	236 (37)	305 (48)	95 (15)
ILC	121	50 (41)	67 (56)	4 (3)
Mixed IDC/ILC	34	18 (53)	13 (38)	3 (9)
Other	34	15 (44)	11 (32)	8 (24)
Grade				
1	92	52 (57)	39 (42)	1 (1)
2	530	226 (43)	263 (49)	41 (8)
3	206	41 (20)	94 (46)	71 (34)
LVI				
+	452	170 (38)	201 (44)	81 (18)
−	338	134 (40)	178 (52)	26 (8)
UK	38	15 (39)	17 (45)	6 (16)
LN Status				
Micrometastases	277	110 (40)	121 (44)	46 (16)
1 LN	378	135 (36)	199 (53)	44 (11)
2 LN	126	54 (33)	56 (44)	16 (13)
3 LN	47	20 (42.5)	20 (42.5)	7 (15)
ENE +				
Yes	296	110 (47)	147 (50)	37 (13)
No	509	200 (39)	239 (47)	70 (14)
UK	23	9 (39)	10 (44)	4 (17)
AJCC staging				
IB	129	59 (46)	49 (38)	21 (16)
IIA	312	111 (36)	163 (52)	37 (12)
IIB	339	132 (39)	159 (47)	46 (14)
IIIA	48	17 (36)	24 (52)	6 (14)
Surgery				
BCS	561	203 (36)	279 (50)	79 (14)
Mastectomy	267	116 (43)	117 (44)	34 (13)
RT received	730	270 (37)	359 (49)	101 (14)
CT received	328	46 (14)	181 (55)	101 (31)

*RS* Recurrence score, *IDC *Invasive ductal carcinoma, *ILC* Invasive lobular carcinoma, *ENE* Extranodal extension, *LVI* Lymphovascular invasion, *BCS *Breast conserving surgery, *RT* Radiotherapy, *CT* Chemotherapy, *LN* Lymph node, *UK* Unknown

Median tumour size was 2.28 cm (range 0.3–12.5 cm). Grade (G)1, G2 and G3 disease was seen in 92 (11%), 530 (64%) and 206 (25%) of patients, respectively. Three-hundred and nineteen (38%) patients had a RS of 0–13, 396 (48%) a RS 14–25 and 113 (14%) a RS > 25. Three-hundred and seventy-eight patients (46%) had 1 lymph node, 126 (15%) 2 lymph nodes and 47 (6%) 3 lymph nodes positive. Micro-metastatic disease only was seen in 277 patients (33.5%).

Mean and median ages were comparable across all test score groups. Similar to our node-negative study (Mc Sorley et al.) [35], the majority of G1 and 2 tumours had an RS result of ≤ 25 (G1; 91/92, 99% and G2; 489/530, 92%). More patients with G3 disease had a RS result of > 25 (71/206; 34%) compared to 41/530 (8%) of patients with G2 disease, and 1/92 (1.1%) of patients with G1 disease.

Breast conserving surgery was performed in the majority of patients, 561 (67.8%), compared with 267 (32.2%) who underwent a mastectomy. Adjuvant radiotherapy was administered to 730 (88%) of patients; of these 270 (37%), 359 (49%) and 101 (14%) had a RS result of 0–13, 14–25 and > 25 respectively. The distribution of RS result was similar regardless of AJCC staging, age < 50 or ≥ 50 years, whether radiotherapy was received, and whether mastectomy or BCS was performed (Table 3).

Tumour grade was the only characteristic linked to increased RS result in women < 50 years, (p < 0.0001). For women ≥ 50 years, both tumour grade (*p* < 0.0001) and multifocality of disease (*p* < 0.0001) were linked to increased RS result.

### Decision impact analysis

Of the 828 patients who underwent Oncotype DX testing, 348 (42%) patients were recommended adjuvant chemotherapy; of these patients, 48 (14%), 191 (55%) and 109 (31%) had RS result of 0–13, 14–25 and > 25, respectively. Nineteen patients refused chemotherapy, and 1 patient was not offered chemotherapy due to comorbidities. Independent of age < 50 years or ≥ 50 years, TC (docetaxel and cyclophosphamide) was the most-commonly prescribed regimen (see appendix). TC was also the most-commonly prescribed regimen for all RS results, except for women < 50 years with RS > 25, for which ACT (adriamycin, cyclophosphamide and paclitaxel) was most frequently used. There was a higher number of patients in our study who received CMF (cyclophosphamide, methotrexate and 5FU) as their adjuvant regimen than currently expected, which may be accounted for in part by the fact that this study includes patients from as far back as 2011. (See appendix).

Post-Oncotype DX testing, 480 patients (58%) had a negative adjuvant chemotherapy recommendation. Of these patients, 446 (54%) were prescribed anti-hormonal therapy alone and 34 (4%) prescribed the addition of ovarian function suppression. Of the patients who had a change in chemotherapy recommendation based on Oncotype DX testing, 271 (56%), 205 (43%) and 4 (1%) had a RS result of 0–13, 14–25 and > 25, respectively. After testing, a change in treatment recommendation in favour of no chemotherapy was seen in 71/92 (77%) of G1, 334/530 (63%) of G2 and 75/206 (36%) of G3 tumours.

### Decision impact analysis and nodal status

No association between the numbers of nodes affected, including micrometastatic vs. macro-metastatic disease, and RS was identified. Of the patients with micrometastatic disease, 103/277 (37%) were recommended adjuvant chemotherapy, alongside 217/378 (57%) of patients with 1 lymph node, 66/126 (52%) of patients with 2 lymph nodes and 19/47 (40%) of patients with 3 lymph nodes post-Oncotype DX testing (Table 4).

### Decision impact in women < 50 years

In total, 171 women aged < 50 years underwent Oncotype DX testing; 54 (32%) had RS 0–13, 89 (52%) RS 14–25 and 28 (16%) RS > 25 (Table 5). Among those with a RS result of 0–13, 23 (43%) were recommended chemotherapy of which 2 patients refused, 61 (69%) with a RS result of 14–25 were recommended chemotherapy of which 1 patient refused, and 27 (96%) with a RS result of > 25 were recommended chemotherapy of which 1 patient refused (Table 4).

**Table 4:** Decision impact analysis according to nodal status and age

Characteristic	Chemotherapy recommended *n* (%)	No Chemotherapy recommended n (%)
*Nodal Status*		
Micrometastases	103 (37)	174 (63)
1 LN	217 (57)	161 (43)
2 LN	66 (52)	60 (48)
3 LN	19 (40)	28 (60)
*Age (yrs)*		
< 50	111 (65)	60 (35)
> 50	237 (36)	420 (64)

**Table 5:** Patients age < 50 presented by tumour grade, Recurrence Score result and chemotherapy (CT) received

	Total	RS 0–13	RS 14–25	RS 26–100
Grade 1	21	11	9	1
Grade 2	103	36	59	8
Grade 3	47	7	21	19
CT	107	21	60	26

With the incorporation of Oncotype DX testing, 111 (65%) women < 50 years of age received a recommendation for adjuvant chemotherapy which resulted in a 35% reduction in chemotherapy use in this patient population (Table 4).

### Decision impact in women ≥ 50 years

Oncotype DX testing was performed in 657 women ≥ 50 years, and 265 (40%) had RS 0–13, 307 (47%) RS 14–25 and 85 (13%) RS > 25. Among those with a RS result of 0–13, 25 (9%) received chemotherapy, 130 (42%) with a RS result of 14–25 were recommended chemotherapy of which 9 patients refused and 82 (96%) with a RS result of > 25 were recommended chemotherapy of which 6 patients refused. One patient was not prescribed chemotherapy due to comorbidities.

Oncotype DX testing resulted in a 64% reduction in chemotherapy use in women ≥ 50 years with 237 (36%) women receiving a post-test chemotherapy recommendation (Table 4).

### Decision impact pre-and post-RxPONDER data release

In our node-positive patient population, changes in the ordering demographics of Oncotype DX tests and chemotherapy prescribing were identified over the time period of our study.

**Patients < 50 years of age.**

The proportion of tests ordered for younger patients was lower during the study period after the RxPONDER study results were reported. The proportion of younger patients who avoided chemotherapy following Oncotype DX testing was also lower during the study period after the RxPONDER study results were reported (Table 6).

**Table 6:** Changes in Oncotype DX ordering practices for time periods 2011–2020 (Pre-RxPONDER) vs. 2021–2022 (Post-RxPONDER)

Age group	Total tests ordered n (%)		Change in treatment decision based on Oncotype DX testing n (%)	
	*Pre-RxPONDER*	*Post-RxPONDER*	*Pre-RxPONDER*	*Post-RxPONDER*
< 50 years	124 (22)	47 (17)	50 (40)	10 (21)
≥ 50 years	433 (78)	224 (83)	261 (60)	159 (71)

**Patients ≥ 50 years of age**

In contrast to the trend observed for the younger patients, the proportion of older patients avoiding chemotherapy following Oncotype DX testing was higher during the study period after the RxPONDER study results were reported (Table 6).

### Budget impact analysis

The use of Oncotype DX testing was associated with a 58% net reduction in chemotherapy administration over the period of this study. This resulted in estimated savings of €6,005,080 in chemotherapy-related costs. Without the use of Oncotype DX testing, the estimated chemotherapy-related costs were €9,944,412. Deducting the assay cost, estimated net savings of €3,372,040 were achieved.

We performed scenario analyses by varying the assumed net reduction in chemotherapy use due to Oncotype DX testing, chemotherapy-related costs and cost of Oncotype DX testing by ± 25%, which did not change the overall conclusions from the analysis that testing was estimated to lead to overall savings (Table 7).

**Table 7:** Scenario analyses of the budget impact calculations

Assumption category	Base case assumptions	Scenarios	Scenario assumptions	Estimated budget impact
Net reduction in use of chemotherapy	50%	+ 25% − 25%	62.5% 37.5%	-€4,873,309 -€1,870,770
Cost per Oncotype DX test	€3,180	+ 25% − 25%	€3,975 €2,385	-€2,713,779 -€4,030,299
Chemotherapy treatment-related costs	€12,010.16	+ 25% − 25%	€15,013 €9,008	-€4,873,460 -€1,870,960

Very few studies to date have included an evaluation of the societal costs associated with chemotherapy for early breast cancer. The importance of this is often overlooked, with direct costs more frequently published as they are more readily calculated. We based the analysis for our study on UK cost estimates from a report by the University of East Anglia, as discussed previously in this article [33]. Using these calculations, the total estimated societal costs of chemotherapy for the patients in this dataset were €100,055,338 without Oncotype DX testing. This figure reduced to €39,635,448 for chemotherapy-related costs with Oncotype DX testing, resulting in an overall saving of €60,419,890 in societal costs.

## Discussion

This large cross-sectional retrospective study provides a real-world evaluation of both the clinical and economic impact of Oncotype DX testing in 828 women with ER-positive, HER2-negative, 1–3 lymph node-positive breast cancer in the Irish Health Service. In our analysis of data from five Irish National Cancer Centres, Oncotype DX testing resulted in a change in treatment recommendation in 58% of patients overall. The change in treatment recommendation was from chemotherapy to anti-hormonal therapy alone, with a small percentage of patients also receiving ovarian function suppression. The change in chemotherapy administration mirrors our previously published study which demonstrated a 62.5% reduction in chemotherapy use in node-negative patients [35].

International decision impact studies conducted across multiple different healthcare systems have demonstrated the clinical utility and economic impact of Oncotype DX testing in N1 patients [21–24, 36–38]. Irrespective of country of origin, a reduction in chemotherapy recommendation of varying magnitude was seen across all studies, ranging from 28 to 69% [21–25, 36–38]. However, since the release of RxPONDER data, our work represents the largest published study which analyses the clinical decision impact and budgetary impact of the use of Oncotype DX testing in N1 patients in a national public health system. Holt et al. reported the results from a large UK study at SABCS in 2022. Their analyses included 664 patients and identified a 65% relative reduction in chemotherapy recommendation, with physicians reporting an increase in confidence in their recommendations after receiving the RS result [39].

The baseline characteristics of our real-world study population compared to RxPONDER are shown in Table 8. The median age and proportion of patients < 50 years are similar between both studies: 57 years and 21% vs. 57.2 years and 24.4% in our study and RxPONDER, respectively. In our study, there is a higher proportion of grade 3 tumours (25% vs. 10.1%), less grade 1 tumours (11% vs. 24.3%), more patients with T2 tumours (50% vs. 36.7%), and less T1 tumours (44% vs. 58.3%) than included in RxPONDER, indicating a potentially higher risk study population consistent with real-world data.

**Table 8:** A comparison of baseline characteristics in our real-world study vs. RxPONDER

	Our analysis (*n* = 828)	RxPONDER^1^ (*n* = 5018)
Median age	57 (22–81)	57.2 (18.3–86)
Age grouping—*n* (%)		
< 50 years	171 (21)	1224 (24.4)
≥ 50 years	657 (79)	3794 (75.6)
LN status—*n* (%)		
1 LN	378 (46)	3275 (65.3)
2 LN	126 (15)	1266 (25.2)
3 LN	47 (6)	460 (9.2)
Not reported	0 (0)	17 (0.3)
21 gene RS—*n* (%)		
0–13	319 (38)	2147 (42.8)
14–25	396 (48)	287 (57.2)
> 25	113 (14)	0 (0)
Tumour Size		
T1	359 (44)	2923 (58.3)
T2	418 (50)	1843 (36.7)
T3	51 (6)	252 (5)
Histological grade		
1	92 (11)	1218 (24.3)
2	530 (64)	3215 (64.3)
3	206 (25)	507 (10.1)
Not reported	0 (0)	15 (0.30)
Histology		
IDC	639 (77)	3673 (74.6)
ILC	121 (15)	674 (13.7)
Mixed IDC/ILC	34 (4)	278 (5.6)
Other	34 (4)	299 (6.1)
Not reported	0 (0)	94 (1.9)
ENE +		
Yes	296 (36)	1051 (21.3)
No/UK	532 (64)	3874 (78.7)

*IDC* Invasive ductal carcinoma, *ILC* Invasive lobular carcinoma, *ENE* Extranodal extension, *LN* Lymph node, *UK* Unknown. (1) Kalinsky K, Barlow WE, Gralow JR, Meric-Bernstam F, Albain KS, Hayes DF, et al. 21-Gene Assay to Inform Chemotherapy Benefit in Node-Positive Breast Cancer. New England Journal of Medicine 2021;385(25):2336–47

Our understanding of the significance of micrometastatic disease with regard to RS risk groupings has been limited by inconsistent categorisation of micrometastatic nodal involvement. In accordance with standard pathological staging practice, some trials include patients with micro-metastases in the LN positive group [10], whereas others have regarded them as LN negative for treatment purposes [40]. In our study, patients with micrometastatic LN involvement represent a significant proportion (37%) of patients who underwent Oncotype DX testing. Similar levels of testing are reported in the literature (43.7–44.4%) [41, 42].

Tumour grade has previously been shown to correlate with adjuvant chemotherapy use most significantly [43]. In this study, higher tumour grade was the only factor that was statistically significant for increased RS result (*p* < 0.0001) in both < 50 and ≥ 50 subgroups, and for women ≥ 50 years, multifocality of disease was also linked to increased RS result (*p* < 0.0001). Within our cohort, the presence of extranodal extension was not significantly associated with RS result (*p* = 0.720). Data published to date on the effect of extranodal extension on RS result are limited but are an area of interest which requires further research [44].

Updated results from RxPONDER confirm initial findings of no additional benefit of chemotherapy in iDFS among postmenopausal women, but a small benefit in some premenopausal women. A post hoc analysis in 200 premenopausal women with micro-metastases identified that chemotherapy provided a 7.3% absolute benefit in iDFS (HR 0.44), and for the 1400 patients with N1 disease, the absolute benefit was 4.8% (HR 0.64) [45]. Our study identifies longitudinal changes in chemotherapy prescribing practices for N1 patients in Ireland pre-and post-RxPONDER results. Over 70% of women ≥ 50 years who had Oncotype DX testing performed after the pivotal RxPONDER trial was published avoided chemotherapy, compared to 60% of women prior to publication of the RxPONDER results. In women < 50 years, findings were different following these results. In our study, we saw a 23% reduction in the proportion of Oncotype DX tests ordered for patients < 50 years after RxPONDER data. We also identified an increase in chemotherapy administration for these patients, where a 40% reduction in chemotherapy administration in the pre-RxPONDER time period fell to 21% post-RxPONDER. This is unsurprising as following RxPONDER the ASCO guidelines were updated to recommend the use of Oncotype DX testing to guide decision making regarding adjuvant chemotherapy use among postmenopausal women with ER-positive, HER2-negative, N1 breast cancer. However, the ASCO 2022 recommendations state that premenopausal women with node-positive tumours should not be offered the Oncotype DX test to guide decisions for adjuvant systemic treatment [46].

Our study demonstrates significant cost savings associated with Oncotype DX testing. Over the course of the study, an estimated saving of €3.3 million was identified for the exchequer. However, in tandem with the economic benefit for the third-party payer, the physiological and financial benefits of avoiding chemotherapy for these women are huge and include but are not limited to emotional wellbeing, avoidance of potential premature mortality and secondary malignancies, and out-of-pocket expenses. On a wider societal scale, chemotherapy leads to significant productivity losses such as short and long-term work absences. Our study estimates over €60 million in societal savings with the use of Oncotype DX testing.

Our study has some limitations. First, this was a retrospective study assessing the impact of the 21-gene score and, therefore, can be prone to recall bias. Second, the treatment decision making without the Oncotype DX test was not recorded at the individual patient level, as this information is not routinely collected. Instead, broad assumptions have been made about the likely chemotherapy treatment decisions for this patient group in the absence of the RS result based on international treatment guidelines.

The decision impact results reflect treatment decisions made based on a discussion with the patient about the Oncotype DX test results in combination with other clinical risk factors.

In conclusion, Oncotype DX testing has led to a clinically meaningful 58% reduction in chemotherapy administration in Irish women with ER-positive, HER2-negative breast cancer with 1–3 positive lymph nodes which has resulted in substantial cost savings for the exchequer. The use of Oncotype DX testing has increased in women ≥ 50 years, which is reflected in the further reduction in chemotherapy administration for these patients post-RxPONDER data release. Continued efforts to identify the subset of premenopausal women who can forgo chemotherapy are now important as confidence has been eroded with the RxPONDER results. As the benefit of chemotherapy in premenopausal women is only relevant for a small proportion of patients, trials such as the Phase III OFSET trial (NCT05879926) [47] which aims to define the survival benefit of the addition of adjuvant chemotherapy to ovarian function suppression plus endocrine therapy among premenopausal patients with ER-positive, HER2-negative tumours, and a RS result between 0 and 25 for N1 patients (and 16–25 for N0 patients) are now a priority.

## Supplementary Information

Below is the link to the electronic supplementary material.

Supplementary file1 (DOCX 17 kb)

## Data Availability

No datasets were generated or analysed during the current study.

## References

[1] Annual Statistical Report 2022: Cancer In Ireland 1994–2022. Available from: https://www.ncri.ie/sites/ncri/files/pubs/NCRI_AnnualStatisticalReport_2022.pdf

[2] Cancer Trends [Internet]. 2019. Available from: https://www.ncri.ie/sites/ncri/files/pubs/Trendsreport_Breastcancer_20191107_0.pdf

[3] Howlader N, Altekruse SF, Li CI, Chen VW, Clarke CA, Ries LAG et al (2014) US incidence of breast cancer subtypes defined by joint hormone receptor and HER2 status. JNCI: J Natl Cancer Inst. 10.1093/jnci/dju055 24777111 10.1093/jnci/dju055 PMC4580552

[4] Aapro M, De Laurentiis M, Rea D, Bargallo Rocha JE, Elizalde R, Landherr L et al (2017) The MAGIC survey in hormone receptor positive (HR+), HER2-negative (HER2-) breast cancer: when might multigene assays be of value? Breast 33:191–199 28441617 10.1016/j.breast.2017.01.012

[5] Early Breast Cancer Trialists’ Collaborative Group (2012) Comparisons between different polychemotherapy regimens for early breast cancer: meta-analyses of long-term outcome among 100 000 women in 123 randomised trials. Lancet 379(9814):432–444 22152853 10.1016/S0140-6736(11)61625-5 PMC3273723

[6] Neri A, Marrelli D, Roviello F, De Stefano A, Guarnieri A, Pallucca E et al (2005) Prognostic value of extracapsular extension of axillary lymph node metastases in T1 to T3 breast cancer. Ann Surg Oncol 12(3):246–253 15827817 10.1245/ASO.2005.02.029

[7] Danko ME, Bennett KM, Zhai J, Marks JR, Olson JA (2010) Improved staging in node-positive breast cancer patients using lymph node ratio: results in 1788 patients with long-term follow-up. J American College Surg 210(5):797-805e1 10.1016/j.jamcollsurg.2010.02.045 20421053

[8] Goldvaser H, Ribnikar D, Majeed H, Ocaña A, Amir E (2018) Absolute benefit from adjuvant chemotherapy in contemporary clinical trials: a systemic review and meta-analysis. Cancer Treat Rev 71:68–75 30366201 10.1016/j.ctrv.2018.10.010

[9] Martin M, Brase JC, Calvo L, Krappmann K, Ruiz-Borrego M, Fisch K et al (2014) Clinical validation of the EndoPredict test in node-positive, chemotherapy-treated ER+/HER2− breast cancer patients: results from the GEICAM 9906 trial. Breast Cancer Res. 10.1186/bcr3642 24725534 10.1186/bcr3642 PMC4076639

[10] Cardoso F, van’t Veer L, Poncet C, Lopes Cardozo J, Delaloge S, Pierga JY et al (2020) MINDACT: long-term results of the large prospective trial testing the 70-gene signature MammaPrint as guidance for adjuvant chemotherapy in breast cancer patients. J Clin Oncol 38:506–516

[11] Harris LN, Ismaila N, McShane LM, Andre F, Collyar DE, Gonzalez-Angulo AM et al (2016) Use of biomarkers to guide decisions on adjuvant systemic therapy for women with early-stage invasive breast cancer: American society of clinical oncology clinical practice guideline. J Clin Oncol 34(10):1134–1150 26858339 10.1200/JCO.2015.65.2289 PMC4933134

[12] Xin L, Liu YH, Martin TA, Jiang WG (2017) The era of multigene panels comes? the clinical utility of oncotype DX and MammaPrint. World J Oncol 8(2):34–40 29147432 10.14740/wjon1019w PMC5649994

[13] Sparano JA, Gray RJ, Makower DF, Pritchard KI, Albain KS, Hayes DF et al (2018) Adjuvant chemotherapy guided by a 21-gene expression assay in breast cancer. N Engl J Med 379(2):111–121 29860917 10.1056/NEJMoa1804710 PMC6172658

[14] Kalinsky K, Barlow WE, Gralow JR, Meric-Bernstam F, Albain KS, Hayes DF et al (2021) 21-gene assay to inform chemotherapy benefit in node-positive breast cancer. N Engl J Med 385(25):2336–2347 34914339 10.1056/NEJMoa2108873 PMC9096864

[15] Paik S, Shak S, Tang G, Kim C, Baker J, Cronin M et al (2004) A multigene assay to predict recurrence of tamoxifen-treated, node-negative breast cancer. N Engl J Med 351(27):2817–2826 15591335 10.1056/NEJMoa041588

[16] Paik S, Tang G, Shak S, Kim C, Baker J, Kim W et al (2006) Gene Expression and benefit of chemotherapy in women with node-negative, estrogen receptor-positive breast cancer. J Clin Oncol 24(23):3726–3734 16720680 10.1200/JCO.2005.04.7985

[17] Recommendations | Early and locally advanced breast cancer: diagnosis and management | Guidance | NICE. www.nice.org.uk. Available at: https://www.nice.org.uk/guidance/ng101/chapter/Recommendations#diagnostic-assessment-and-adjuvant-therapy-planning. Accessed January 11, 2024

[18] NCCN Clinical Practice Guidelines in Oncology (NCCN Guidelines®) Breast cancer Version 1. 2016. The National Comprehensive Cancer Network. Available at: www.nccn.org. 2016. Accessed January 11, 2024

[19] Albain KS, Barlow WE, Shak S, Hortobagyi GN, Livingston RB, Yeh IT et al (2010) Prognostic and predictive value of the 21-gene recurrence score assay in postmenopausal women with node-positive, oestrogen-receptor-positive breast cancer on chemotherapy: a retrospective analysis of a randomised trial. Lancet Oncol 11(1):55–65 20005174 10.1016/S1470-2045(09)70314-6 PMC3058239

[20] Stemmer SM, Steiner M, Rizel S, Geffen DB, Nisenbaum B, Peretz T et al (2017) Clinical outcomes in ER+ HER2 -node-positive breast cancer patients who were treated according to the recurrence score results: evidence from a large prospectively designed registry. NPJ Breast Cancer. 10.1038/s41523-017-0033-7 28900632 10.1038/s41523-017-0033-7 PMC5591314

[21] Eiermann W, Rezai M, Kümmel S, Kühn T, Warm M, Friedrichs K, Schneeweiss A, Markmann S, Eggemann H, Hilfrich J et al (2013) The 21-gene recurrence score assay impacts adjuvant therapy recommendations for ER-positive, node-negative and node-positive early breast cancer resulting in a risk-adapted change in chemotherapy use. Ann Oncol 24:618–624 23136233 10.1093/annonc/mds512 PMC3574549

[22] Dieci MV, Guarneri V, Zustovich F, Mion M, Morandi P, Bria E, Merlini L, Bullian P, Oliani C, Gori S et al (2019) Impact of 21-gene breast cancer assay on treatment decision for patients with T1–T3, N0–N1, estrogen receptor-positive/human epidermal growth receptor 2-negative breast cancer: final results of the prospective multicenter ROXANE study. Oncologist 24:1424–1431 31152079 10.1634/theoncologist.2019-0103 PMC6853101

[23] Chin-Lenn L, De Boer RH, Segelov E, Marx GM, Hughes TM, McCarthy NJ, White SC, Foo SS, Rutovitz JJ, Della-Fiorentina S et al (2018) The impact and indications for oncotype DX on adjuvant treatment recommendations when third-party funding is unavailable. Asia Pac J Clin Oncol 14:410–416 30270527 10.1111/ajco.13075

[24] Loncaster J, Armstrong A, Howell S, Wilson G, Welch R, Chittalia A, Valentine WJ, Bundred NJ (2017) Impact of oncotype DX breast recurrence score testing on adjuvant chemotherapy use in early breast cancer: real world experience in Greater Manchester UK. Eur J Surg Oncol 43:931–937 28111076 10.1016/j.ejso.2016.12.010

[25] Berdunov V, Laws E, Cuyun Carter G, Luo R, Russell C, Campbell S et al (2023) The budget impact of utilizing the oncotype DX breast recurrence score test from a US healthcare payer perspective. J Med Economics 26(1):973–990 10.1080/13696998.2023.2235943 37466220

[26] A rapid health technology assessment of gene expression profiling tests for guiding the use of adjuvant chemotherapy in early- stage invasive breast cancer. (2023). Available at: https://www.hiqa.ie/sites/default/files/2023-02/HTA%20of%20GEP%20tests%20to%20guide%20treatment%20in%20early%20breast%20cancer_Full%20report.pdf. Accessed August 8, 2024

[27] ABF 2022 Admitted Patient Price List DRG Prices for Inpatients and Daycases 2022. Available from: https://www.hpo.ie/abf/ABF2022AdmittedPatientPriceList.pdf. Accessed December 12, 2023

[28] Smyth L, Watson G, Walsh EM, Kelly CM, Keane M, Kennedy MJ et al (2015) Economic impact of 21-gene recurrence score testing on early-stage breast cancer in Ireland. Breast Cancer Res Treat 153(3):573–582 26364296 10.1007/s10549-015-3555-4

[29] HSE Primary Care Reimbursement Service (PCRS) list of high-tech medicines. January 2019. Available from: https://www.hse.ie/eng/staff/pcrs/online-services/jan-2019-list-of-prescribably-high-tech-medicines.pdf. Accessed September 15, 2023

[30] Health Information and Quality Authority. A rapid health technology assessment of gene expression profiling tests for guiding the use of adjuvant chemotherapy in early- stage invasive breast cancer [Internet]. 2023. Available from: https://www.hiqa.ie/sites/default/files/2023-02/HTA%20of%20GEP%20tests%20to%20guide%20treatment%20in%20early%20breast%20cancer_Full%20report.pdf. Accessed September 16, 2023

[31] Ellis PD, Barrett-Lee P, Johnson LC, Cameron D, Wardley AM, O’Reilly SE et al (2009) Sequential docetaxel as adjuvant chemotherapy for early breast cancer (TACT): an open-label, phase III, randomised controlled trial. Lancet 373(9676):1681–1692 19447249 10.1016/S0140-6736(09)60740-6 PMC2687939

[32] Carney P, O’Boyle D, Larkin A, McGuigan C, O’Rourke K (2018) Societal costs of multiple sclerosis in Ireland. J Med Econ 5:425–437 10.1080/13696998.2018.1427100 29320900

[33] Hinde S, Theriou C, May S, Matthews L, Arbon A, Fallowfield L et al (2019) The cost-effectiveness of EndoPredict to inform adjuvant chemotherapy decisions in early breast cancer. Health Policy Technol 8(1):75–83

[34] Parsekar K, Howard Wilsher S, Sweeting A, Patel A, Fordham R (2021) Societal costs of chemotherapy in the UK: an incidence-based cost-of-illness model for early breast cancer. BMJ Open 11(1):e039412. 10.1136/bmjopen-2020-039412 33431487 10.1136/bmjopen-2020-039412 PMC8728345

[35] McSorley LM, Tharmabala M, Al Rahbi F, McSorley K, Chew S, Evoy D et al (2021) Real-world analysis of clinical and economic impact of 21-gene recurrence score (RS) testing in early-stage breast cancer (ESBC) in Ireland. Breast Cancer Res Treat 188(3):789–798 33835293 10.1007/s10549-021-06211-w

[36] Hassan S, Younan R, Patocskai E, Provencher L, Poirier B, Sideris L, Dubé P, Boileau J-F, Mihalcioiu C, Robidoux A (2021) Abstract PS4-27: A prospective multicenter study evaluating the impact of the 21-gene breast recurrence score® upon physician treatment decision and cost in lymph node-positive breast cancer patients in Quebec. Cancer Res. 10.1158/1538-7445.Sabcs20-ps4-2733795251

[37] Mattar A, Fonseca GR, Romão MBA, Shida JY, de Oliveira VM, Bastos MCS, Bagnoli F, Rinaldi JF, Stiepcich M, da Silva M et al (2021) Substantial reduction in adjuvant chemotherapy with the use of the 21-gene test to manage early breast cancer in a public hospital in Brazil. JCO Glob Oncol 7:1003–1011 34181482 10.1200/GO.20.00609 PMC8457870

[38] Gomez HL, Bargallo-Rocha JE, Billinghurst RJ, Núñez De Pierro AR, Coló FA, Gil LLB, Allemand C, McLean IL, Lema-Medina M, Herazo-Maya F et al (2021) Practice-changing use of the 21-gene test for the management of patients with early-stage breast cancer in Latin America. JCO Glob Oncol 7:1364–1373 34506221 10.1200/GO.21.00008 PMC8440019

[39] Holt S, Verrill M, Pettit L et al (2024) A UK prospective multicentre decision impact, decision conflict and economic evaluation of the 21-gene assay in women with node+ve, hormone receptor+ve, HER2-ve breast cancer. Br J Cancer 130:1149–1156. 10.1038/s41416-024-02588-9 38308000 10.1038/s41416-024-02588-9 PMC10991515

[40] Dowsett M, Cuzick J, Wale C, Forbes J, Mallon EA, Salter J et al (2010) Prediction of risk of distant recurrence using the 21-gene recurrence score in node-negative and node-positive postmenopausal patients with breast cancer treated with anastrozole or tamoxifen: A TransATAC study. J Clin Oncol 28(11):1829–1834 20212256 10.1200/JCO.2009.24.4798

[41] Peethambaram PP, Hoskin TL, Day CN et al (2017) Use of 21-gene recurrence score assay to individualize adjuvant chemotherapy recommendations in ER+/HER2−node positive breast cancer—a national cancer database study. NPJ Breast Cancer 3:1–9. 10.1038/s41523-017-0044-4 29067357 10.1038/s41523-017-0044-4 PMC5648884

[42] Roberts MC, Miller DP, Shak S, Petkov VI (2017) Breast cancer-specific survival in patients with lymph node-positive hormone receptor-positive invasive breast cancer and Oncotype DX recurrence score results in the SEER database. Breast Cancer Res Treat 163:303–310. 10.1007/s10549-017-4162-3 28243896 10.1007/s10549-017-4162-3

[43] Nguyen MT, Stessin A, Nagar H, D’Alfonso TM, Chen Z, Cigler T et al (2014) Impact of oncotype DX recurrence score in the management of breast cancer cases. Clin Breast Cancer 14(3):182–190 24486121 10.1016/j.clbc.2013.12.002

[44] Jasem J, Fisher CM, Amini A et al (2017) The 21-gene recurrence score assay for node-positive, early-stage breast cancer and impact of RxPONDER trial on chemotherapy decision-making: have clinicians already decided? J Natl Compr Cancer Netw 15:494–503. 10.6004/jnccn.2017.0049 10.6004/jnccn.2017.0049 28404760

[45] Kalinsky KM, Barlow WE, Gralow JR, et al: Distant disease-free interval in participants with 1–3 positive lymph nodes, hormone receptor-positive and HER2-negative breast cancer with a recurrence score < or = 25 randomized to endocrine therapy +/- chemotherapy SWOG S1007 (RxPONDER) y: Presented at the 2021 San Antonio Breast Cancer Symposium (SABCS). Absract GS2–07.

[46] Andre F, Ismaila N, Allison KH, Barlow WE, Collyar DE, Damodaran S et al (2022) Biomarkers for adjuvant endocrine and chemotherapy in early-stage breast cancer: ASCO guideline update. J Clin Oncol 40(16):1816–1837 35439025 10.1200/JCO.22.00069

[47] Evaluating the Addition of Adjuvant Chemotherapy to Ovarian Function Suppression Plus Endocrine Therapy in Premenopausal Patients With pN0-1, ER-Positive/HER2-Negative Breast Cancer and an Oncotype Recurrence Score Less Than or Equal to 25. ClinicalTrials.gov. Available from: https://classic.clinicaltrials.gov/ct2/show/NCT05879926. Accessed January 14, 2023

