# Supplementary material for: Irish national real-world analysis of the clinical and economic impact of 21-gene oncotype DX® testing in early-stage, 1-3 lymph node-positive, oestrogen receptor-positive, HER2-negative, breast cancer
Source: Breast Cancer Res Treat. 2024 Oct 4;209(1):189–99. doi: 10.1007/s10549-024-07486-5 (PMC11785674; doi:10.1007/s10549-024-07486-5)
Supplement: Supplementary file 1 — Supplementary file1 (DOCX 17 kb) [file 10549_2024_7486_MOESM1_ESM.docx]

**Table 1: Chemotherapy selection in patients aged < 50 by Recurrence Score result**

| Chemotherapy Total | | Recurrence Score | | |
| --- | --- | --- | --- | --- |
|  | | 0-13 | 14-25 | >25 |
| AC x 4 | 1 | 0 | 1 | 0 |
| ACT | 40 | 5 | 21 | 14 |
| CMF | 15 | 3 | 12 | 0 |
| 4 x CMF – 12 T | 3 | 0 | 1 | 2 |
| T x 12 | 2 | 1 | 1 | 0 |
| TC x 4 | 32 | 8 | 18 | 6 |
| TC x 6 | 9 | 3 | 4 | 2 |
| TC-AC | 2 | 0 | 1 | 1 |
| Unknown | 3 | 1 | 1 | 1 |
| Declined | 4 | 2 | 1 | 1 |
| Total | 111 | 23 (21%) | 61 (55%) | 27 (24%) |

TC, Taxotere/cyclophosphamide; AC, Adriamycin/cyclophosphamide; AC-T, Adriamycin/cyclophosphamide – paclitaxel; CMF, cyclophosphamide/methotrexate/fluorouracil; T, paclitaxel

**Table 2: Chemotherapy selection in patients aged ≥ 50 by Recurrence Score result**

| Chemotherapy Total | | Recurrence Score | | |
| --- | --- | --- | --- | --- |
|  | | 0-13 | 14-25 | >25 |
| AC x 4 | 4 | 1 | 3 | 0 |
| ACT | 37 | 3 | 10 | 24 |
| CMF | 53 | 4 | 37 | 12 |
| T x 12 | 7 | 3 | 2 | 2 |
| TC x 4 | 78 | 10 | 47 | 21 |
| TC x 6 | 35 | 4 | 19 | 12 |
| TC-AC | 2 | 0 | 1 | 1 |
| Unknown | 5 | 0 | 2 | 3 |
| Declined | 15 | 0 | 9 | 6 |
| Not given (comorbidities) | 1 | 0 | 0 | 1 |
| Total | 237 | 25 (10%) | 130 (55%) | 82 (35%) |

TC, Taxotere/cyclophosphamide; AC, Adriamycin/cyclophosphamide; AC-T, Adriamycin/cyclophosphamide – paclitaxel; CMF, cyclophosphamide/methotrexate/fluorouracil; T,paclitaxel

‌
